# New roles for “old” microRNAs in nervous system function and disease

**DOI:** 10.3389/fnmol.2013.00051

**Published:** 2013-12-24

**Authors:** Marion Hartl, Ilona C. Grunwald Kadow

**Affiliations:** ^1^MRC Clinical Science Center, Hammersmith Hospital CampusLondon, UK; ^2^Max-Planck Institute of NeurobiologyMartinsried, Germany

**Keywords:** *let-7*, *bantam*, *miR-279*, nervous system, development, behavior, regeneration, degeneration

## Abstract

Since their discovery, microRNAs became prominent candidates providing missing links on how to explain the developmental and phenotypical variation within one species or among different species. In addition, microRNAs were implicated in diseases such as neurodegeneration and cancer. More recently, the regulation of animal behavior was shown to be influenced by microRNAs. In spite of their numerous functions, only a few microRNAs were discovered by using classic genetic approaches. Due to the very mild or redundant phenotypes of most microRNAs or their genomic location within introns of other genes many regulatory microRNAs were missed. In this review, we focus on three microRNAs first identified in a forward genetic screen in invertebrates for their essential function in animal development, namely *bantam*, *let-7*, and *miR-279*. All three are essential for survival, are not located in introns of other genes, and are highly conserved among species. We highlight their important functions in the nervous system and discuss their emerging roles, especially during nervous system disease and behavior.

## INTRODUCTION

The discovery of microRNAs was a big step towards the understanding of post-transcriptional regulation of gene expression. Several hundreds of microRNAs capable of interacting with a plethora of target mRNAs were discovered in model organisms. While sequence based prediction tools of microRNA-target interaction evolved quickly, phenotypical analysis of microRNA function lagged behind, in part due to the lack of clear phenotypes in mutants of single microRNAs. In addition, phenotypes were expressed only under certain environmental and experimental conditions suggesting that microRNAs act predominately by fine-tuning gene expression. As a consequence, microRNAs were rarely discovered in genetic screens. However, three microRNAs were found in genetic forward screens in invertebrates. All three are essential for survival, are not located in introns of other genes, and are highly conserved among species.

## INITIAL DISCOVERY OF *Let*-7, *Bantam*, AND *miR*-279

*Let-7* was the first microRNA described in *C. elegans*. It is conserved across different species and found in all common model organisms. In *C. elegans* *let-7* participates in the so-called heterochronic pathway, which regulates the transition between different developmental stages in the worm by timing the division and differentiation of stem cells. *Let-7* is upregulated in the last larval stage (L4), and by downregulating *lin-41* mRNA, allows the animal to fully mature. Weak mutant alleles of *let-7* lead to a reiteration of larval patterns of cell division, and the animal fails to differentiate. Strong alleles of *let-7* mutants cause a severe phenotype of a blasting vulva (Reinhart et al., 2000). The second example, *bantam*, has no mammalian homolog and has been intensively studied in *Drosophila*. Mutations of this microRNA affect the proliferation of the wing disk and lead to failure in the G1-S transition of wing disk cells (Brennecke et al., 2003; Herranz et al., 2008). The third microRNA, *miR-279*, is highly conserved in insects. The first phenotype described was found in the olfactory system of *Drosophila*. In this system, *miR-279* regulates the differentiation of a subclass of olfactory receptor neurons by downregulating two transcription factors, Nerfin-1 and Escargot (Cayirlioglu et al., 2008; Hartl et al., 2011).

Here, we review recent advances in the understanding of the function of *let-7*, *bantam*, and *miR-279* in neural development, regeneration and degeneration, and behavior.

## *Let*-7 REGULATES CELLULAR DIFFERENTIATION OF THE NMJ AND OTHER BRAIN STRUCTURES

*In silico* analysis predicted a strong interaction of *let-7* with the transcription factor Abrupt. Recent studies verified this microRNA-target relationship in the neuromuscular junction (NMJ) and the mushroom body (MB) in *Drosophila*. In both tissues, the microRNA controls the developmental transition to an adult shape. When missing, the NMJ retains a juvenile shape and is not able to fully differentiate (Sokol et al., 2008). Although no anatomical phenotype was detected, *let-7* mutant flies show defects in locomotion, flight, and also fertility (Sokol et al., 2008). The authors could show that the phenotype is accompanied by increased levels of the broad-complex, tramtrack, and bric-a-brac (BTB) transcription factor Abrupt in *let-7* mutants (Caygill and Johnston, 2008) corroborating a previous finding that Abrupt ensures the remodeling of the larval NMJ to achieve its adult shape and function (Hu et al., 1995).

Two other recent studies in *Drosophila* show that the *let-7* complex (*let-7-C*) is a key regulator of the development of the MB (Kucherenko et al., 2012; Wu et al., 2012). *Let-7-C* gives rise to three different microRNAs, namely (*let-7, miR-100,* and *miR-125*), which can act individually but also synergistically on mRNA regulation. The *Drosophila* MB is a complex brain structure essential for olfactory learning and memory as well as context-dependent innate behavior (Heisenberg, 2003; Fiala, 2008; Bracker et al., 2013). It is comprised of Kenyon cells (KCs) that can be further classified into four different subtypes. During development, they are derived from multi-potent progenitor cells and are born in a fixed order (γ→α′/β′→pioneer α/β→α/β; Zhu et al., 2003, 2006). Two factors guiding the precise timing of MB neuron subtypes are the transcription factors, “Chronologically inappropriate morphogenesis” (Chinmo) and Abrupt (Zhu et al., 2006; Kucherenko et al., 2012). Chinmo affects the differentiation of MB subtypes in a concentration dependent manner. While high levels of Chinmo in post-mitotic neurons specify γ and α′/β′, low levels of Chinmo drive the differentiation of late-born MB neurons (pioneer α/β, α/β; Zhu et al., 2006). In order to generate different levels of Chinmo throughout MB development, *let-7* and *miR-125* co-transcribed from the *let-7-C locus,* contribute to the progressive downregulation of *chinmo* *in vivo*. *Let-7* and *miR-125* regulate *chinmo* expression directly via binding sites in the 3′UTR of the transcription factor. The third microRNA of the *let-7-C, mir-100*, seems not to be involved in the post-transcriptional regulation (Wu et al., 2012). All *let-7-C* microRNAs are strongly upregulated in the transition from the late pupal to early adult stage. *In vivo*, precocious expression of *let-7* and *miR-125* in larval stage 1 leads to a sharp decrease of Chinmo levels already in larval stage 3. As a consequence, the adult MB shows strong morphological defects and mis-differentiation of its cell types. The second study revealed that *let-7-C* also influences the timing of α′/β′ to α/β transition via the Chinmo related BTB transcription factor Abrupt (Kucherenko et al., 2012). The differentiation of the late born α/β neurons depends on the expression of *let-7C*. By contrast, Abrupt is essential to establish the identity of α′/β′ neurons. Thus, *let-7-C* mediated downregulation of Abrupt regulates the transition between different subsets of MB neurons. Notably, the expression of *let-7-C* appears to be dependent on Ecdysone signaling, a key regulator for morphological transitions during insect development (Robbins et al., 1968). While both studies describe effects on MB morphology, the effect of the *let-7-C* mutation on MB morphology differs in the two studies. Wu et al. only find delays in the transition towards different MB subtypes. By contrast, Kucherenko et al. detect a significant reduction of α/β lobe volume. Reasons for these phenotypical differences are not known, but may have to do with the role of microRNAs as buffers rather than instructors of gene expression. Therefore different experimental conditions such as nutrition and temperature might influence the severity of the phenotype. Nevertheless, the results of the two publications show that *let-7-C* is used to sharpen the expression of two potent transcription factors in order to produce different neuronal subtypes. While Chinmo has a broader effect on MB development and seems to affect the generation of all MB subtypes, Abrupt only affects the transition of the late born neurons.

## MECHANISMS OF *Let*-7 REGULATION

In order to precisely time the expression of potent transcription factors during development, microRNA expression needs to be tightly regulated in expression. To ensure precise timing of activity for instance during neuronal differentiation, *let-7* interacts with one of its classical targets in an autoregulatory cycle. In embryonic stem (ES) and embryonic carcinoma (EC) cells, the pluripotency factor Lin-28 binds *pre-let-7* and inhibits the last step during *let-7* processing and thereby prevents the formation of a mature microRNA** (Rybak et al., 2008). In neural stem cells, *lin-28* is repressed by *let-7* and *miR-125.* This leads to a neural stem cell commitment towards differentiated neurons (Rybak et al., 2008).

The transcription factor SRY (sex-determining region)-box 2 (SOX2) directly binds the Lin-28 promotor and regulates its expression (Cimadamore et al., 2013). Expression and activation of Lin-28 inhibits *let-7* and leads to the maturation of the NPCs, which are derived from human ES cells (Cimadamore et al., 2013). In this context, loss of SOX2 as well as overexpression of *let-7* (specifically of *let-7i*) led to the inhibition of neuronal differentiation (Cimadamore et al., 2013).

## *Bantam* DETERMINES CELLULAR GROWTH IN THE NERVOUS SYSTEM

During development, two processes must be coordinated: first, cells differentiate to obtain their cellular fate and function, and second, the cell number is multiplied forming the basis of growth and proliferation. Recent studies implicate the microRNA *bantam* in glia proliferation in the *Drosophila* brain and optic lobe. *Drosophila* larvae undergo an extreme growth in the third instar stage. Epidermal growth is often linked to the well-studied Hippo pathway. In a recent study, Reddy and Irvine (2011) show an involvement of the pathway in non-epithelial glia cells for the first time. In the Hippo pathway, Merlin acts as the upstream regulator of the core kinase cascade in the Hippo pathway. Merlin depletion or expression of an activated form of Yorkie leads to glia overgrowth in the optic lobe and the brain of *Drosophila*. Similar to the wing disk, Merlin activates the expression of Yorkie and in turn, Yorkie was found to activate the expression of *bantam* (Nolo et al., 2006; Thompson and Cohen, 2006). As a consequence of *bantam*, expression levels of Myc are increased, probably as an indirect effect due to suppression of the ubiquitin ligase Mei-P26 (Herranz et al., 2010). Interestingly, neurons are insensitive to increased levels of *bantam* and hence, glia cells are affected exclusively.

Another study describes a role for *bantam* during the development of another neural structure, the optic lobe. *Bantam* is highly expressed in mitotically active cells in the developing optic lobe. Depletion of *bantam* levels in third instar *Drosophila* larvae leads to smaller optic lobes, whereas overexpression of *bantam* results in an increased volume of optic lobes. The effect of *bantam* is not due to a mis-differentiation of glia cells since even in the full mutant larvae glia cells are present. In this context *bantam* seems to influence the number of glia cells. The authors conclude that *bantam* maintains the pool of stem cells during development and thus influences the proliferation of the cells. In the same study, the T-box transcription factor Omb was identified as downstream target of *bantam* that can also partially rescue the gain-of-function phenotype of the microRNA. The molecular mechanism of how *bantam* controls the cell cycle remains to be investigated (Li and Padgett, 2012).

## *miR*-279 AS A MOLECULAR SWITCH OF CO_2_ NEURON LOCATION

*miR-279* is highly conserved in all insect species, and was first identified in a forward genetic screen for axon guidance and synapse formation factors using the olfactory system of *Drosophila* as a model system (Cayirlioglu et al., 2008). The olfactory system in *Drosophila* consists of two appendages, the antenna and the maxillary palp, which house different sets of olfactory sensory neurons (OSNs). During development each subset of OSNs sends their axons to a defined area, a glomerulus, in the antennal lobe in the brain to generate a spatial representation of odors. In the olfactory system, *miR-279* regulates development and axon targeting of a specific class of sensory neurons. Flies mutant for *miR-279* develop extra CO_2_ sensory neurons on the second olfactory appendage of insects, the maxillary palp, in addition to CO_2_ neurons found on control antenna (Jones et al., 2007; Kwon et al., 2007). These ectopic CO_2_ neurons innervate a glomerulus in the antennal lobe associated with the detection of food cues and not with the detection of CO_2_ (Cayirlioglu et al., 2008) The innervation pattern of the ectopic CO_2_ neurons highly resembles the location and central brain innervation of mosquito CO_2_ neurons (Ghaninia et al., 2007; Lu et al., 2007). Therefore, *miR-279* was proposed as a molecular switch in the divergence of mosquitoes and flies for the trait of CO_2_ detection (Cayirlioglu et al., 2008; Jones, 2008). Since flies are highly repelled by CO_2_ and mosquitoes strongly attracted to it the study provides a starting point to explore how molecular changes in regulation shape neural circuits and thereby the behavioral output (Benton, 2008; Jones, 2008). Similar to *let-7,* *miR-279* appears to regulate neuronal commitment and differentiation of progenitor cells (Cayirlioglu et al., 2008; Hartl et al., 2011; **Table 1**). On the mechanistic level, *miR-279* expression is regulated by the pan-neuronal transcription factor Prospero (Hartl et al., 2011), an important player in stem cell progression and sensory neuron development (Doe et al., 1991; Choksi et al., 2006). Two transcription factors both of which are also direct targets of Prospero, the snail transcription factor Escargot and the zinc-finger transcription factor Nerfin-1 were identified as essential and sufficient targets of *miR-279*: gain-of-function of both targets together induced the formation and mistargeting of ectopic CO_2_ neurons efficiently (Hartl et al., 2011). Thus, the pan-neural factor Prospero refines its own activity by inducing a microRNA to regulate the expression of its own downstream target genes. Given Prospero’s role during stem cell development in both flies and mice a use of the same or similar network is conceivable also in the control of tumor formation in the brain or the lymphatic system (Petrova et al., 2002; Galeeva et al., 2007).

**Table 1:** Conserved functions of *let-7*, *bantam, and miR-279* in the nervous system.

	microRNA	Model organism	Function	Citation
Development	*let-7*	*Drosophila*	Maturation of neuromuscular junction (NMJ)	Caygill and Johnston (2008), Sokol et al. (2008)
			Mushroom body (MB) differentiation	Kucherenko et al. (2012), Wu et al. (2012)
		Human neural precursor cells embryonic stem cells	Pluripotency	Rybak et al. (2008), Cimadamore et al. (2013)
	*bantam*	*Drosophila*	Glia cell growth in the brain and optic lobe	Reddy and Irvine (2011)
			Differentiation and number of glia cells in the opticlobe	Li and Padgett (2012)
	*miR-279*	*Drosophila*	CO_2_ neuron development	Cayirlioglu et al. (2008), Hartl et al. (2011)
Regeneration	*let-7*	*C. elegans*	AVM neuron axon regeneration	Zou et al. (2013)
		Zebrafish	De-differentiation of Mueller glia cells	Ramachandran et al. (2010)
	*bantam*	*Drosophila*	Dendritic aborisation (da) neuron regeneration	Song et al. (2012)
Degeneration	*let-7*	Mouse	Loss of cortical neurons through extracellular *let-7*	Lehmann et al. (2012)
Behavior	*miR-279*	*Drosophila*	Regulation of circadian rhythm	Luo and Sehgal (2012)

## NEURONAL REGENERATION

**FIGURE 1:**
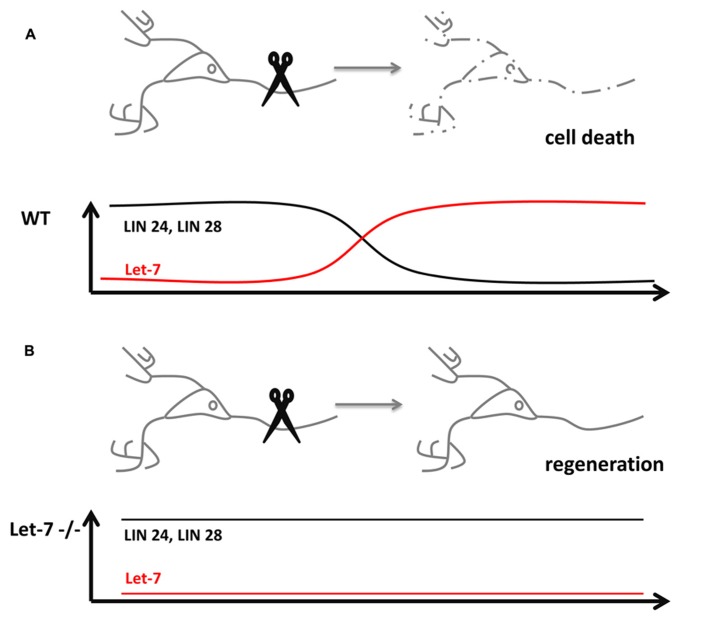
**microRNAs influence neuronal regeneration. **(A)** Severing of an axon usually causes cell death in wildtype neurons (dashed lines), which lost its potential for regeneration through the downregulation of the pluripotency factors Lin 24 and 28 by microRNA *let-7*. **(B)***let-7*^-^^/^^-^ neurons are still able to regenerate destroyed axons, since the pluripotency factors Lin 24 and 28 stay expressed even in post-mitotic cells**.

While aging, the nervous system progressively loses the ability to rapidly regenerate new cells. The decline in regeneration during aging is a conserved phenomenon. In *C. elegans,* the anterior ventral microtubule (AVM) axon is used to study the effect of single molecules on the regeneration of neurons. The regeneration decline in *C. elegans* occurs usually in the larval stage 3 (L3) prior to the transition to young adults. Worms mutant for xmicroRNA biosynthesis factors Dicer-1 or Argonaute Alg-1, however, continue to regenerate the axons of AVM which in turn extend much longer as compared to wildtype controls (Zou et al., 2013). In this context, the microRNA involved and responsible for increased axonal length and regeneration is *let-7* (**Figure 1**). Mutants of *let-7* exhibited the same phenotype in AVM neurons as *alg-1* mutants. The study showed that in order to stop AVM axons from extending, Lin-41 is strongly repressed by *let-7* in late adult stages. Mutants of *lin-41* show a decreased regeneration of the axons. Interestingly,** Lin-41 co-immunoprecipitates with Alg-1, which constitutes a key factor for *let-7* biogenesis. The experiments suggest that in early stages of development a Lin-41/Alg-1 complex is formed and represses the synthesis of *let-7* permitting axonal regeneration and extension. In late larval up to the young adult stages, this suppression is removed and *let-7* is processed to the mature microRNA, which effectively downregulates *lin-41*. In turn, the AVM axons are no longer able to regenerate and stop to grow. Therefore, the molecular mechanism underlying the regeneration decline of AVM axons exemplifies, how a regulatory circuit is reused in post-mitotic cells.

Another example of how a pluripotency factor is used to trigger regeneration in post-mitotic cells was studied in the fish retina. The retina of fish has the remarkable potential to fully regenerate after injury. In order to recover, Müller glia cells de-differentiate and form new progenitors. The regeneration potential is based on the high expression of the pluripotency factor Lin-28 in de-differentiated Müller glia cells, a feature shared with ES cells (Rybak et al., 2008; **Table 1**). Since Lin-28 is also a known target of *let-7*, the fish retina exemplifies the autoregulatory mechanism of the microRNA and its target in a regeneration inducing process (Ramachandran et al., 2010).

In another system, the dendritic arborisation (da) neurons found in the body wall of *Drosophila* larvae, microRNA *bantam* was found to be involved in the process of balancing the growth of dendrites and the underlying epithelium as the target area of the neurons. During a process called scaling, the microRNA functions as a signal to synchronize the neuronal growth with the epithelium. When *bantam* is missing, the dendrites overshoot and fail to cover the appropriate space. Interestingly, during this process, *bantam* is not expressed in the neurons but in the epithelial target cell and acts as a signal to downregulate Akt signaling in the neurons. How the microRNA signal is transferred is not yet found and opens up a new field of study dealing with microRNA signal transduction via direct microRNA transport between neighboring cells (Parrish et al., 2009). In a new study, the axons and dendrites of one class of da neurons are established as a model for neuronal regeneration (Song et al., 2012). Similar to the mammalian system, peripheral sensory neurons retain the potential to regenerate after injury, whereas the processes of central neurons fail to regenerate after injury. Studies how to overcome this lack of regeneration were mostly performed in the mammalian system. However, this study shows in detail that the da neurons in the *Drosophila* body wall and the ventral nerve cord can serve as a model to identify the molecular players involved in regeneration.

Not only in the axons, also da neuron dendrites in *bantam* mutants regenerate. The effect could also be mimicked through depletion of PTEN or gain-of-function of Akt in the neurons. Because Akt signaling is involved in scaling as well as in regeneration of the da neurons, regulatory cycles important during are likely reused during regeneration.

## *Let*-7 IS INVOLVED IN THE DEGENERATION OF NEURONS

**FIGURE 2:**
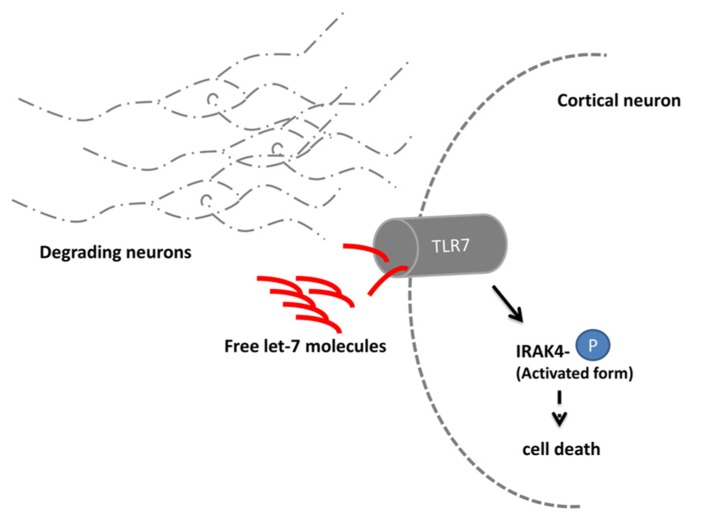
***let-7* regulates neuronal degeneration. Neurons degrading due to e.g., the consequences of Alzheimer’s disease release the microRNA *let-7*. Extracellular elevated *let-7* molecules are sensed by the Toll-like receptor 7 (TLR 7) which is expressed in cortical neurons. Upon binding the downstream molecule IRAK 4 becomes phosphorylated and induces through yet unidentified signaling molecules cell death**.

An unexpected role of *let-7* was revealed in a study on signaling mechanisms leading to neuronal degeneration (Lehmann et al., 2012). Upon a neuronal damage, e.g., during the course of Alzheimer’s disease, the immune system multiplies the degradation process through a so far unidentified mechanism. In a recent study, the RNA sensing receptor Toll-like 7 (TLR 7) in cortical neurons of mice was shown to bind extracellular enriched *let-7* released by degenerating neurons (**Figure 2**). Subsequently, the TLR 7 expressing cell undergoes apoptosis. Mice injected with *let-7b* into the spinal canal lose 18% of neurons after 3days in the cortical area where TLR 7 is endogenously expressed. A comparable loss of neurons occurred also in the striatal area of the mouse brain. After 2weeks, the effect further increased up to a loss of 30% of neurons. In mice lacking the TLR 7 receptor, no neuronal loss was detectable. Injection of *let-7b* was in turn also sufficient to activate downstream TLR 7 signaling which was shown by the increased phosphorylation state of IRAK4 (Takeuchi and Akira, 2010).

Moreover, an increase of extracellular *let-7b* was also measured in the corticospinal fluid (CSF) of Alzheimer patients (Lehmann et al., 2012). This observation implies a conserved function of the microRNA as signaling molecule in neurodegenerative diseases. Whether depletion of *let-7b* could decrease the extent of neurodegeneration in Alzheimer disease patients appears to be an interesting hypothesis to be tested in future studies.

## microRNAs CONTROL BEHAVIOR

**FIGURE 3:**
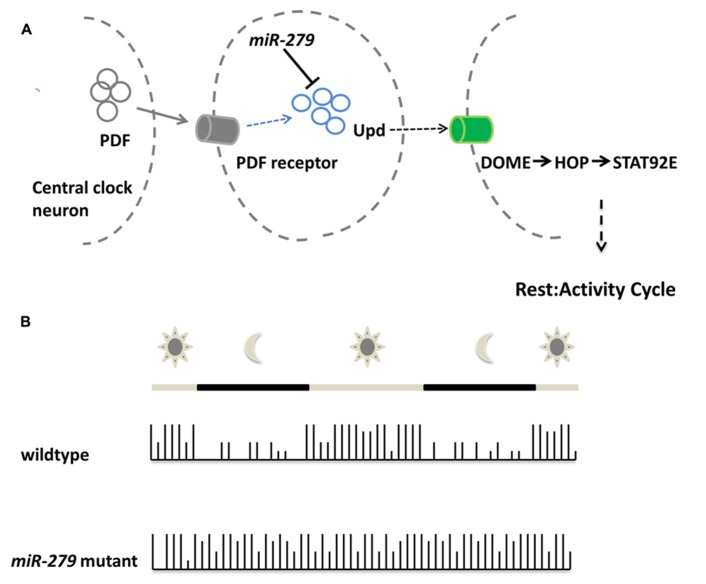
***miR-279* regulates the circadian rhythm in *Drosophila.***(A)**Schematic showing the molecular mechanisms of central clock neurons to downstream neurons in order to regulate the rest/activity cycle. The central clock neurons release PDF which is sensed via the pdf receptor in target cells. Upon PDF receptor activation, the expression of the cytokine Unpaired is activated, but also repressed via the microRNA *miR-279*. The released cytokine is bound by the receptor DOME in central clock downstream neurons which eventually assure the transition between rest and activity in the circadian rhythm of the fly. **(B)** During the day a wildtype fly shows alternating patterns of activity and rest. During daytime the fly shows high activity which is indicated by the high bars in the activity plot. During the night the rest cycle starts, indicated by low bars. The microRNA-279 mutant flies do not show rest/activity cycles anymore and show a overall activity throughout day and night**.

Recent work implicated microRNA function also in the behavior of adult animals. Using an overexpression screen, Luo and Sehgal (2012) demonstrated that *miR-279* controls the circadian rhythm of flies (**Figure 3**). Enhanced levels of *miR-279* were found to disrupt the rest-activity cycles of *Drosophila*. Cycles in circadian rhythm are linked to the oscillations of Period in so-called pacemaker neurons (Ozkaya and Rosato, 2012). These oscillations are normal in *miR-279* mutants suggesting that the microRNA is an effector of Period transmitting signals from pacemaker neurons. Interestingly, *miR-279* targets the secreted morphogen unpaired (Upd) using a JAK/STAT-dependent interaction that was recently discovered in the oocyte (Yoon et al., 2011). The *Drosophila* ovaries contain migratory border cells and non-migratory follicle cells. A gradient of the secreted morphogen unpaired (Upd) is used to establish the cell fate of the two subtypes (Yoon et al., 2011). In order to activate JAK/STAT signaling, the cytokine Upd is secreted and binds STAT (signal transducer and activator of transcription). High levels of Upd specify migratory border cells (Silver and Montell, 2001; Ghiglione et al., 2002), whereas low or transient levels specify the non-migratory follicle cells (Starz-Gaiano et al., 2008). In the oocyte, *miR-279* was found to favor the cell fate of follicle cells through repression of STAT. In the adult fly, reducing the levels of Upd in *miR-279* mutants rescues the circadian rhythm phenotype. Central pacemaker neurons target neurons positive for Upd and were proposed to be candidates for signal receivers of the central clock. This study shows for the first time, which signaling pathway transmits the PER protein oscillations of the circadian clock to downstream neurons. In addition, it represents one of the rare examples for a function of microRNAs in behavior without affecting the development of the underlying circuits.

## DISCUSSION

Mutants of only a few microRNAs were found to be essential or to exhibit substantial phenotypes. Of the characterized microRNAs, most appear to fine-tune expression of target mRNAs. The microRNAs with a quantifiable and persistent phenotype are good candidates to study microRNA function and regulation in detail. Three microRNAs, *let-7*, *bantam,* and *miR-279* were discovered in forward genetic screens, because their mutants showed substantial developmental phenotypes. Notably, these microRNAs are encoded by their own genetic loci, in contrast to microRNAs that are found in intronic regions of other genes. In this review, we focused on the roles of these well studied microRNAs in the nervous system. Besides multiple developmental effects of these microRNAs, more recent studies find them to be involved in regeneration, degeneration, and also behavior.

During development the discussed microRNAs target very often pluripotency factors to ensure the proper transition between differentiation and proliferation of different cell types. In mutants, cells fail to differentiate or properly grow. Interestingly, the same microRNA target relationships also affect regeneration events in post-mitotic neurons or glia cells. As exemplified in the fish retina and fly da neurons blocking the expression of *let-7* or *bantam* enables the cell to increase the levels of pluripotency factors to allow cell growth and thereby regeneration of cells and tissues.

In contrast, *let-7* uses a new and different mechanism in the context of neuronal degeneration. Here, an increase in *let-7* levels in degenerating neurons induces cell death of cortical neurons. In this case, *let-7* acts as an extracellular signaling molecule. Two very interesting questions arise from this finding: (i) how are microRNAs transported? and (ii) do other microRNAs work as signaling molecules? The type of microRNAs and the mechanisms of microRNA-mediated extracellular signaling might also be of interest in light of a potential use of microRNAs as therapeutic agents to prevent neuronal cell loss in neurodegenerative diseases such as Alzheimer’s (Chen et al., 2012). We also discussed a study of a novel role of *miR-279* during the circadian rhythm regulation in *Drosophila*. This study provided an interesting example of the function of small regulatory RNAs in the adult animal behavior. The authors demonstrated that *miR-279* helps transmitting the JAK/STAT pathway signal to neurons downstream of pace maker neurons without affecting their development. This study is remarkable, because it showed for the first time that oscillations of Period levels are transmitted to downstream neurons. Regarding the role of microRNAs, the work encourages a search for additional microRNAs involved in the regulation of behavior.

However, the cases of the three discussed microRNAs also imply that the approach of identifying microRNAs through a forward screen approach has weaknesses, because of the highly redundant function of the majority of these molecules. Through the development and further refinement of RNAseq methods, it is now possible to identify the set of microRNAs expressed in a specific tissue or even in a single cell. Alternatively to forward genetic screens, RNAseq mediated analysis of expression of all microRNAs in a given sample could be used to specifically manipulate a combination of microRNAs. These manipulations are being facilitated by the existence of new methods such as site-directed excision with Zinc finger nucleases (Zfn), TALENs (transcription activator-like effector nuclease), or CRISPR-Cas (clustered regulatory interspaced short palindromic repeat; Gaj et al., 2013). Using a modified reverse genetic approach the function of a specific microRNA or groups of microRNAs could be revealed.

Thanks to the work of many groups, we have gained a substantial understanding of microRNA function during development of the nervous system and beyond. The recent findings that microRNAs regulate aspects of adult animal biology such as neuronal degeneration and regeneration as well as behavior suggest that still much is to be discovered to complete our picture of microRNA-mediated gene regulation and signaling. New genetic and genomic approaches and constantly improved methods to monitor gene expression will pave the way to analyze the function not only of single essential microRNAs but also of groups of microRNAs during nervous system development and in the adult animal.

## Conflict of Interest Statement

The authors declare that the research was conducted in the absence of any commercial or financial relationships that could be construed as a potential conflict of interest.
